# Trismus Nascentium

**Published:** 1877-08

**Authors:** J. N. Dixon

**Affiliations:** Springfield, Ills.


Trismus Nascentium.—*By J. N. Dixon, M.D., Springfield, Ills.* On the 3d day of February I was invited by Dr. Rowler to see with him an interesting case of trismus nascentium. Upon our arrival, found the patient, a fine and well developed male child, ten days old, suffering from a well marked case of trismus, with spasms recurring every ten or fifteen minutes, increasing in frequency and severity. The jaws were firmly locked, and the lips slightly separated and pressed firmly against the gums; the angles of the mouth were drawn backwards and downwards; pulse hardly perceptible; skin warm and perspiring; abdomen slightly swollen and tympanitic.

Dr. R. informed me that he first saw the patient on the evening of February 1st, and since then the child has neither slept nor taken a particle of nourishment of any kind. The treatment had been purgation, emesis, the hot bath, local application of ice to the spine, and tincture physostigma, with the usual routine of constitutional treatment; but without any appreciable benefit. Prognosis—Evidently cannot live many hours.

Having had no experience in the treatment of this disease, and knowing that our most eminent authors recommend the above treatment, I was at a loss to suggest any plan that would benefit our little patient; but remembering of having read an article a few years ago in a medical journal—the name of which I cannot now recall—on the local application of chloroform to the spine for the relief of spasms, and as a last resort, I suggested to Dr. R. giving it a trial. The experiment—for experiment it was—was considered worthy of a trial. I would here state, that with the treatment already detailed, the *inhalation* of chloroform had been thoroughly tried, and found to afford relief only when *total* anæsthesia was produced, and only so long as this condition was sustained. As I said before, the experiment was considered worthy of a trial, and the application was made by saturating a strip of soft cotton cloth with chloroform, and applying it to the entire length of the spine, just as the approach of another paroxysm was announced. As a result of the application, the paroxysm was averted, and in less than fifteen minutes our little patient fell into a quiet sleep, which lasted nearly two hours. Anxious to watch the effects and influence of the chloroform thus applied, we remained with our little patient several hours, during which time several paroxysms were averted by the timely and continuous application of chloroform. For the next thirty-six hours there were occasional tetanic symptoms, which, however, promptly and invariably yielded to the application of the chloroform. The subsequent convalescence was rather slow, but entirely satisfactory.

So satisfied was I with the results of the treatment of this case, and so anxious was I to have another opportunity of testing its efficacy, that I waited very impatiently indeed; but at last it came.

On the 31st of May I delivered Mrs. McB. of a healthy and well developed male child at full term. Both mother and child progressed as usual until June 6th, when I was called to see the child. The mother stated that for the last ten hours it was with great difficulty that the child nursed, although appearing anxious to nurse, and eagerly pressing its mouth against the nipple; at the same time it continued to utter a whimpering, whining, unnatural cry. While the mother was describing the symptoms, I diagnosed the case trismus, but still wanted the pathognomonic symptom, spasms. My diagnosis was soon verified, for before I left the house the child had a violent paroxysm, which lasted several minutes. As soon as possible I applied a strip of cloth, saturated with chloroform, to the entire spine, covering it immediately with oiled silk to prevent evaporation. The result was indeed gratifying. The spasms were promptly and completely arrested, and in a few minutes the child fell into a quiet, calm, and seemingly natural sleep, which lasted three hours. Ordered that the application be applied immediately whenever there was the slightest indications of a return of the paroxysms. Invariably upon the subsidence of the burning pain immediately incident to the application of the chloroform, and which lasted only a few minutes, the child would fall into a peaceful and quiet sleep. The application was continued in this manner for three days, when I was compelled to discontinue it for a time and apply emollients, on account of the vesication produced. During the application of the emollients, a recurrence of the paroxysms was carefully guarded against by the cautious administration of chloroform by inhalation. Of course attention was given to sustaining the child with nourishment, and the bowels were kept open with mild laxatives. At the expiration of forty-eight hours I re-applied the chloroform to the spine, alternating it with inhalation and the emollient applications when necessary.

The convalescence was rapid, and without any unfavorable symptoms.

These two cases are of course too limited to predicate a treatment upon, from which universal and unfailing success is to be expected, but from the unprecedented success thus far resulting from local anæsthesia in the treatment of trismus nascentium, I cannot but think that those who may have occasion or opportunity to give it a fair and impartial trial in the treatment of this exceedingly distressing and generally fatal disease, will have no occasion to be dissatisfied with the results.—*Ohio Medical Recorder.*

